# High Seroprevalence of SARS-CoV-2 in White-Tailed Deer (Odocoileus virginianus) at One of Three Captive Cervid Facilities in Texas

**DOI:** 10.1128/spectrum.00576-22

**Published:** 2022-03-23

**Authors:** Christopher M. Roundy, Chase M. Nunez, Logan F. Thomas, Lisa D. Auckland, Wendy Tang, Jack J. Richison, Breanna R. Green, Clayton D. Hilton, Michael J. Cherry, Alex Pauvolid-Corrêa, Gabriel L. Hamer, Walter E. Cook, Sarah A. Hamer

**Affiliations:** a Department of Entomology, Texas A&M University, College Station, Texas, USA; b Department of Veterinary Pathobiology, Texas A&M University, College Station, Texas, USA; c Department of Veterinary Integrative Biosciences, Texas A&M University, College Station, Texas, USA; d Caesar Kleberg Wildlife Research Institute, Texas A&M University-Kingsville, Kingsvillle, Texas, USA; Changchun Veterinary Research Institute

**Keywords:** SARS-CoV-2, captive cervid, coronavirus, spillover, white-tailed deer, zoonosis

## Abstract

Free-ranging white-tailed deer (Odocoileus virginianus) across the United States are increasingly recognized for infection and transmission of SARS-CoV-2. Through a cross-sectional study of 80 deer at three captive cervid facilities in central and southern Texas, we provide evidence of 34 of 36 (94.4%) white-tailed deer at a single captive cervid facility seropositive for SARS-CoV-2 by neutralization assay (PRNT_90_), with endpoint titers as high as 1,280. In contrast, all tested white-tailed deer and axis deer (*Axis axis*) at two other captive cervid facilities were seronegative, and SARS-CoV-2 RNA was not detected in respiratory swabs from deer at any of the three facilities. These data support transmission among captive deer that cannot be explained by human contact for each infected animal, as only a subset of the seropositive does had direct human contact. The facility seroprevalence was more than double of that reported from wild deer, suggesting that the confined environment may facilitate transmission. Further exploration of captive cervids and other managed animals for their role in the epizootiology of SARS-CoV-2 is critical for understanding impacts on animal health and the potential for spillback transmission to humans or other animal taxa.

**IMPORTANCE** As SARS-CoV-2 vaccine coverage of the human population increases and variants of concern continue to emerge, identification of the epidemiologic importance of animal virus reservoirs is critical. We found that nearly all (94.4%) of the captive white-tailed deer at a cervid facility in central Texas had neutralizing antibodies for SARS-CoV-2. This seroprevalence is over double than that which has been reported from free-ranging deer from other regions of the United States. Horizontal transmission among deer may be facilitated in confinement. Tracking new infections among wild and confined deer is critical for understanding the importance of animal reservoirs for both veterinary and human health.

## INTRODUCTION

Despite many public health preventative measures and the advance of vaccination worldwide, epidemic waves of severe acute respiratory syndrome coronavirus 2 (SARS-CoV-2) caused by more transmissible and antibody evasive genetic variants of concern have sustained the ongoing virus circulation (1, 2). The susceptibility to infection and potential capacity to transmit SARS-CoV-2 in domestic and wildlife species have been increasingly reported (3–6); this understanding is especially critical given wild animals remain unvaccinated and could serve as viral reservoirs.

Recent experimental studies and reports of natural infection have demonstrated that the white-tailed deer (Odocoileus virginianus) is highly susceptible to infection and can transmit SARS-CoV-2 by contact and vertical transmission (7–11). Notably, animals that were infected by contact shed infectious virus in nasal secretions for up to 7 days, indicating that SARS-CoV-2 can be propagated for prolonged periods in a herd after a single infection (7). Approximately 37% to 40% seroprevalence of wild white-tailed deer to SARS-CoV-2 was reported across three Midwestern states (8) and Texas (12). Additionally, one third of 283 free-living and captive white-tailed deer from Iowa sampled during the pandemic had SARS-CoV-2 RNA in their lymph nodes including several lineages circulating in humans, suggesting multiple spillover events followed by deer-to-deer transmission (13). In Ohio, over one third (35.8%) of 360 wild deer had SARS-CoV-2 positive nasal swabs comprised of several variants, including those that were dominate as well as uncommon in the human population (14).

The captive cervid industry arose from growing demand for deer and deer products and involves the raising, or containment propagation of native and non-native cervids in privately maintained facilities, often involving close human-deer contacts. The operations of captive cervid facilities are diverse and include venison and antler production, sales to other cervid operations, coordinated and uncoordinated propagation, providing for commercial fenced hunting preserves, genetic enhancement of private deer herds, and more. There are approximately 10,000 deer breeding and/or hunting facilities in North America (15), most of which include white-tailed deer. The industry is particularly important in Texas where the deer hunting and breeding industry generates over $1.6 billion in economic activity annually (16). Given the recent findings of wild white-tailed deer infection with SARS-CoV-2, combined with the precedent from mink that captive animal facilities may be high risk transmission environments (17–19), our objective was to investigate the SARS-CoV-2 exposure and infection status of captive cervids in Texas.

## RESULTS

Between September 15, 2021 and November 29, 2021, we collected and tested respiratory and rectal swabs and serum of 80 deer from three captive cervid facilities in central and southern Texas. Across all 80 deer from all facilities, respiratory and rectal swabs from the white-tailed deer and fallow deer were RT-qPCR negative. At facility A, 34 of 36 (94.4%) of adult white-tailed deer (mean age of 3.5 years among bucks, 4.5 years for does) were seropositive for SARS-CoV-2 with endpoint titers as high as 1,280 (Table 1); a subset of the does only had direct human contact. At facilities B and C, all white-tailed and fallow deer were seronegative.

**TABLE 1:** Diagnostic details of captive cervids tested for SARS-CoV-2 RNA and antibodies at three facilities in central and South Texas, September 2021 to October 2021

Site	County	Species	Sex	No. tested	RT-qPCR positives		PRNT90		
					Respiratory Swab	Rectal swab	Seropositives (%)	Titer range	Titer geometric mean
Facility A	Guadalupe, TX	Odocoileus virginianus	F	21	0	0	19 (90.50%)	20 to 320	69.13
			M	15	0	0	15 (100%)	20 to 1280	152.77
Facility B	Montgomery, TX	*Axis*	F	8	0	0	0 (0%)	<10	<10
			M	7	0	0	0 (0%)	<10	<10
		*Dama dama*	M	1	0	0	0 (0%)	<10	<10
Facility C	Kleberg, TX	Odocoileus virginianus	F	14	0	0	0 (0%)	<10	<10
			M	15	0	0	0 (0%)	<10	<10

## DISCUSSION

We found that nearly all (94.4%) the tested white-tailed deer at a single captive cervid facility in Texas were positive for SARS-CoV-2 neutralizing antibodies, yet they were negative for viral RNA in respiratory and rectal swabs. All the tested deer at two other captive cervid facilities were seronegative and negative for viral RNA. Although clinical data were not systematically recorded for research purposes, the seropositive deer were observed at least once daily and were not reported to show any clinical signs of disease at any time prior to, during, or after they were sampled.

The time point of deer exposure to SARS-CoV-2 remains unknown. However, considering that antibody persistence in deer is unclear, all swabs tested negative by RT-qPCR, and deer have been shown to shed viral RNA for at least 22 days (9), it would be reasonable to assume the exposure occurred between the beginning of the pandemic to over a month before sampling. Therefore, seropositive deer could have been potentially exposed to any of the 11 most important variants of SARS-CoV-2 that have circulated in the U.S, prior to Omicron (20). Considering that neutralizing antibodies mounted for recently emerged variants are apparently capable to neutralize ancestor lineages (21), but the opposite may not be true in all cases (22), the use of an early lineage of SARS-CoV-2 for the PRNT was providential for minimizing the occurrence of false seronegatives.

All the fallow deer (*Dama dama*) at facility B were among the negative deer. Fallow deer are an exotic species in Texas that now free-range and occur in captivity across much of the state. Despite the absence of previous information regarding SARS-CoV-2 infection in these exotic deer, a closely related species (*Dama gazelle*) was shown based on *in silico* analysis to have medium conservation properties of 25 amino acids important for the binding between ACE2 and the SARS-CoV-2 spike protein (23), suggesting susceptibility to SARS-CoV-2 infection. Future studies of sympatric native and exotic cervids would allow insight into the potential for cross-cervid transmission within captive facilities or the wild.

Many, but not all, of the seropositive animals were used previously in research to evaluate an experimental anthrax vaccine. Nineteen of the 21 white-tailed does tested at facility A were either given experimental vaccine or the commercially available Sterne spore vaccine, while all of the 15 white-tailed bucks tested were neither researched or handled by humans prior to sampling. Furthermore, many of the seronegative individuals from facility C were enrolled in the same anthrax vaccine immunogenicity study and showed no titers to SARS-CoV-2, indicating that cross-reactivity from the various anthrax vaccines is unlikely.

The level of exposure among deer at the captive cervid facility (94.4%) is more than double that which has recently been reported across several studies of free-ranging individuals (8, 12–14); one explanation may be that onward transmission among deer is facilitated by the confined environment. The circulation of SARS-CoV-2 among captive animals could result in eventual spillover to other wildlife species with unknown impact for the conservation and public health, considering the high rates of mutation and recombination observed among coronaviruses (24). A One Health approach is critical to advance the understanding of the potential impact of human-animal interface for SARS-CoV-2 transmission, maintenance, and evolution.

## MATERIALS AND METHODS

### Captive cervid facilities.

Between September 2021 and October 2021, three captive cervid facilities in Texas were sampled. Facility A is a private white-tailed deer breeding and hunting ranch in Guadalupe County, TX, with approximately 207 captive adult white-tailed deer (57 bucks, 150 does) used for breeding and research purposes; 21 of these does were previously enrolled in experimental anthrax vaccine work that involved being handled individually for <1 min for blood collection at approximately monthly time points from November 2020 to July 2021. The facility includes 13 pens ranging from 0.4 to 1.6 acres; 12 for housing breed stock and one for housing research animals, all of which have fence line contact with two to five occupied deer pens. Once-a-day scheduled feedings in each pen were provided by either the ranch foreman or owner. Facility B is a private 7.2-acre pen in Montgomery County, TX, housing approximately 22 Axis deer (*Axis axis*; nine bucks, 13 does) and seven fallow deer (*Dama dama*; two bucks, five does) for breeding purposes. The exotic cervids’ ages ranged from 0.5 to 7.5 years. Human contact consisted of approximately once a week feeder checks by one of five individuals. Facility C consisted of six deer pens ranging from 0.3 to 1.1 acres and contained a research deer herd owned by Texas A&M University-Kingsville in Kleberg County, TX, with approximately 40 white-tailed deer (19 bucks, 21 does) ranging in age from 0.5 to 13.5 years used for teaching and research. Approximately 25 of the deer from facility C had been previously enrolled in the same anthrax vaccine research as facility A and all, except the eight fawns from 2021, have previously been involved in ecology and management studies.

### Sample collection.

During the course of regular veterinary care of deer under physical restraint or chemical immobilization, samples were collected for the SARS-CoV-2 investigation. Two swabs were collected from deer using polyester-tipped applicators with polystyrene handles (Puritan Medical Products, Guilford, ME, USA) and stored in viral transport media (VTM; made following CDC SOP#: DSR-052-02): (i) respiratory (which consisted of one nasal swab and one oral swab placed together into the vial) and (ii) rectal. Blood samples were collected using jugular venipuncture into no-additive or clot activator tubes. All samples were collected in accordance with the relevant guidelines and regulations approved by the TAMU’s Institutional Animal Care and Use Committee and Clinical Research Review Committee (2018-0460 CA).

### Molecular diagnostics.

Aliquots of VTM supernatant were subjected to RNA/DNA extraction by MagMAX CORE Nucleic acid purification kit (Thermo Fisher Scientific, Waltham, MA). An aliquot of purified nucleic acid was tested for SARS-CoV-2 RNA by specific real time RT-qPCR to amplify the RdRp gene (25); a control plasmid containing a portion of the RdRp gene served as a positive control (Integrated DNA Technologies, Coralville, IA, USA). Using this same protocol, our lab has successfully completed the USDA COVID proficiency testing exercise (ICE-2) in the summer of 2021 (26).

### Plaque reduction neutralization tests.

Serum samples were tested by plaque reduction neutralization tests (PRNT) to quantify antibodies able to neutralize the formation of SARS-CoV-2 plaques on Vero CCL-81 cell cultures following standard protocols (27) in a Biosafety Level 3 laboratory. Serum samples were heat inactivated and screened at a dilution of 1:10 and those that neutralized SARS-CoV-2 viral plaques by at least 90%, when compared with the virus control, were further tested at serial 2-fold dilutions starting at 1:10 to determine 90% endpoint titers. Infectious viral stocks used for PRNT were prepared with SARS-CoV-2 Isolate USAIL1/2020, NR 52381 (BEI Resources, Manassas, VA, USA).
